# Identification of putative master regulators in rheumatoid arthritis synovial fibroblasts using gene expression data and network inference

**DOI:** 10.1038/s41598-020-73147-4

**Published:** 2020-10-01

**Authors:** Naouel Zerrouk, Quentin Miagoux, Aurelien Dispot, Mohamed Elati, Anna Niarakis

**Affiliations:** 1 GenHotel, Univ. Évry, Université Paris-Saclay, 91025 Genopole, Évry France; 2 University Lille, CNRS, Inserm, CHU Lille, Centre Oscar Lambret, UMR9020, UMR1277, Canther, Cancer Heterogeneity, Plasticity and Resistance To Therapies, 59000 Lille, France

**Keywords:** Bioinformatics, Gene expression analysis, Diseases, Rheumatoid arthritis, Mechanisms of disease

## Abstract

Rheumatoid arthritis (RA) is a systemic autoimmune disease that affects the synovial joints of the body. Rheumatoid arthritis fibroblast-like synoviocytes (RA FLS) are central players in the disease pathogenesis, as they are involved in the secretion of cytokines and proteolytic enzymes, exhibit invasive traits, high rate of self-proliferation and an apoptosis-resistant phenotype. We aim at characterizing transcription factors (TFs) that are master regulators in RA FLS and could potentially explain phenotypic traits. We make use of differentially expressed genes in synovial tissue from patients suffering from RA and osteoarthritis (OA) to infer a TF co-regulatory network, using dedicated software. The co-regulatory network serves as a reference to analyze microarray and single-cell RNA-seq data from isolated RA FLS. We identified five master regulators specific to RA FLS, namely BATF, POU2AF1, STAT1, LEF1 and IRF4. TF activity of the identified master regulators was also estimated with the use of two additional, independent software. The identified TFs contribute to the regulation of inflammation, proliferation and apoptosis, as indicated by the comparison of their differentially expressed target genes with hallmark molecular signatures derived from the Molecular Signatures Database (MSigDB). Our results show that TFs influence could be used to identify putative master regulators of phenotypic traits and suggest novel, druggable targets for experimental validation.

## Introduction

Rheumatoid arthritis (RA) is a chronic autoimmune disease that primarily affects the synovial joints, but can also cause systemic disorders of cardiovascular, pulmonary, and skeletal nature^1^. While the precise aetiology of RA is still unknown, its pathogenesis involves a combination of genetic background, regarding the presence of susceptibility genes, and environmental triggers, like smoking or the presence of periodontal disease, for example. The characteristic symptoms consist of synovial inflammation, swelling and deformity of the joints, as well as redness, joint stiffness and pain, cartilage damage and bone destruction. The disease affects 0.5 to 1% of the population and appears more frequently in females than males in a 3:1 ratio with the onset settled approximately after 40 years of age^2^. Autoantibodies detected in RA patients include the rheumatoid factor (RF) and the anti-citrullinated protein antibodies (ACPA). RF is an autoantibody that targets the Fc portion of immunoglobulin G (IgG), and its presence correlates with bone erosion and a poorer prognosis. Similarly, the ACPA-positive subset of RA has a more aggressive clinical phenotype compared to ACPA-negative one^3,4^. The early stage of RA starts with leukocyte infiltration of the synovial compartment. Then, the synovial fluid is inundated with pro-inflammatory mediators inducing an inflammatory cascade (synovitis). During this stage, fibroblast-like synoviocytes (FLS) interact with the cells of the innate (monocytes, macrophages, mast cells, dendritic cells) and the adaptive immunity (T cells and B cells). The fulminant stage of the disease is characterized by hyperplastic synovium, cartilage damage, bone erosion, and systemic consequences. TNFα and IL-6 are pro-inflammatory cytokines and two of the primary mediators of RA. Drugs targeting these cytokines are considered leading therapies in RA, and a growing number of them have been commercialized. However, despite their relative success in RA treatment, 40% of patients fail to adequately respond to therapy (response rate of approximately 20%), and only a minority achieves remission in the short term, while for many these periods of disease remission are followed by flare-ups and progression^5,6^.

In healthy joints, the synovial membrane consists of the intimal lining layer called intima, with a thickness of approximately two cells, and a distinct synovial sublining layer, called sub-intima. The resident cells of the synovial membrane are fibroblast-like synoviocytes (FLS) and macrophage-like synoviocytes (MLS). The intima comprises of a layer of FLS and MLS while the sub-intima includes scattered blood vessels, fat cells, and FLS, with few lymphocytes or MLS. In RA, inflammation and cellular infiltration cause hyperplasia of the synovial tissue leading to the pannus formation. The pannus is a tissue composed of MLS, FLS, leucocytes, plasma cells and mastocytes that mediates cartilage damage and bone erosion^7–10^ .

In RA, fibroblast-like synoviocytes (FLS) play a significant role in the initiation and perpetuation of destructive joint inflammation. RA FLS express immuno-modulating cytokines, a variety of adhesion molecules and matrix remodelling enzymes. These cells display tumour-like characteristics like anchorage-independent growth, resistance to apoptosis, and elevated rates of cellular proliferation. In addition, they can cause damage by invading and attacking the adjacent cartilage and bone. Therefore, FLS-targeted therapies could be proposed as complementary approaches to immune-directed treatments to alleviate the debilitating symptoms of the disease^11–13^.

Advancements in high throughput technologies have allowed an unprecedented wealth of information regarding gene expression, even in a single cell scale. This type of data can be used in computational biology approaches to facilitate the reconstruction of gene regulatory networks. Gene expression data reflect functional stages of the analyzed samples and can help determine the dominant factors that drive the cells towards a specific phenotype. To elucidate the complexity of gene regulation and expression, identifying key transcription factors that control and coordinate these processes, is an essential step towards a deeper understanding of disease pathogenesis and progression.

In the present study, we aim to characterize TFs active in RA FLS, in comparison to fibroblasts from patients suffering from osteoarthritis (OA), a degenerative, non-autoimmune disease of the joints. We seek to identify TFs that behave as master regulators and are, therefore, responsible for the regulation and gene expression in RA FLS.

**Figure 1:**
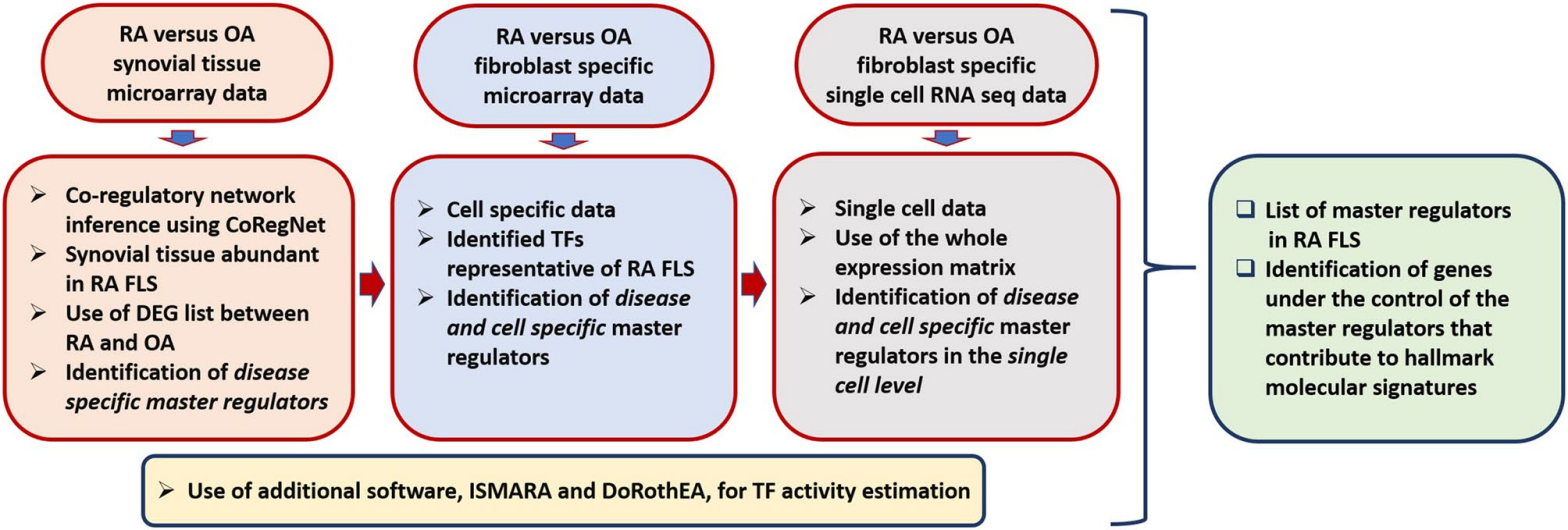
Overview of the analysis workflow for identifying master regulators in RA FLS using *omic* data, network inference and TF activity estimation.

Computational inference of TF activities based on putative target genes using expression data is constantly gaining ground as a way to obtain mechanistic and functional insights from the transcriptome. This abstraction of the transcriptome through TFs activity reduces the number of features, and allows for the identification of master regulators responsible for different cellular phenotypes^14–25^. In our approach, we use publicly available transcriptomic data of synovial tissue and the tool CoRegNet^24^ to infer a reference co-regulatory network. This network provides the basis for detecting the most influential TFs, first in RA synovial tissue, and then in the RA FLS. To improve cell specificity of the master regulators identified, we use microarray and scRNA-seq data from freshly isolated RA FLS (Fig. 1). Two additional tools, DoRothEA^20^ and ISMARA^21^ are employed to estimate independently the activity of the TFs identified with CoRegNet. Lastly, we compare the differentially expressed target genes of the identified TFs, with hallmark molecular signatures derived from Msig^26^, to characterize the TFs influence on the cellular phenotype.

## Results

### Putative co-regulatory network inference for the RA synovial tissue

**Figure 2:**
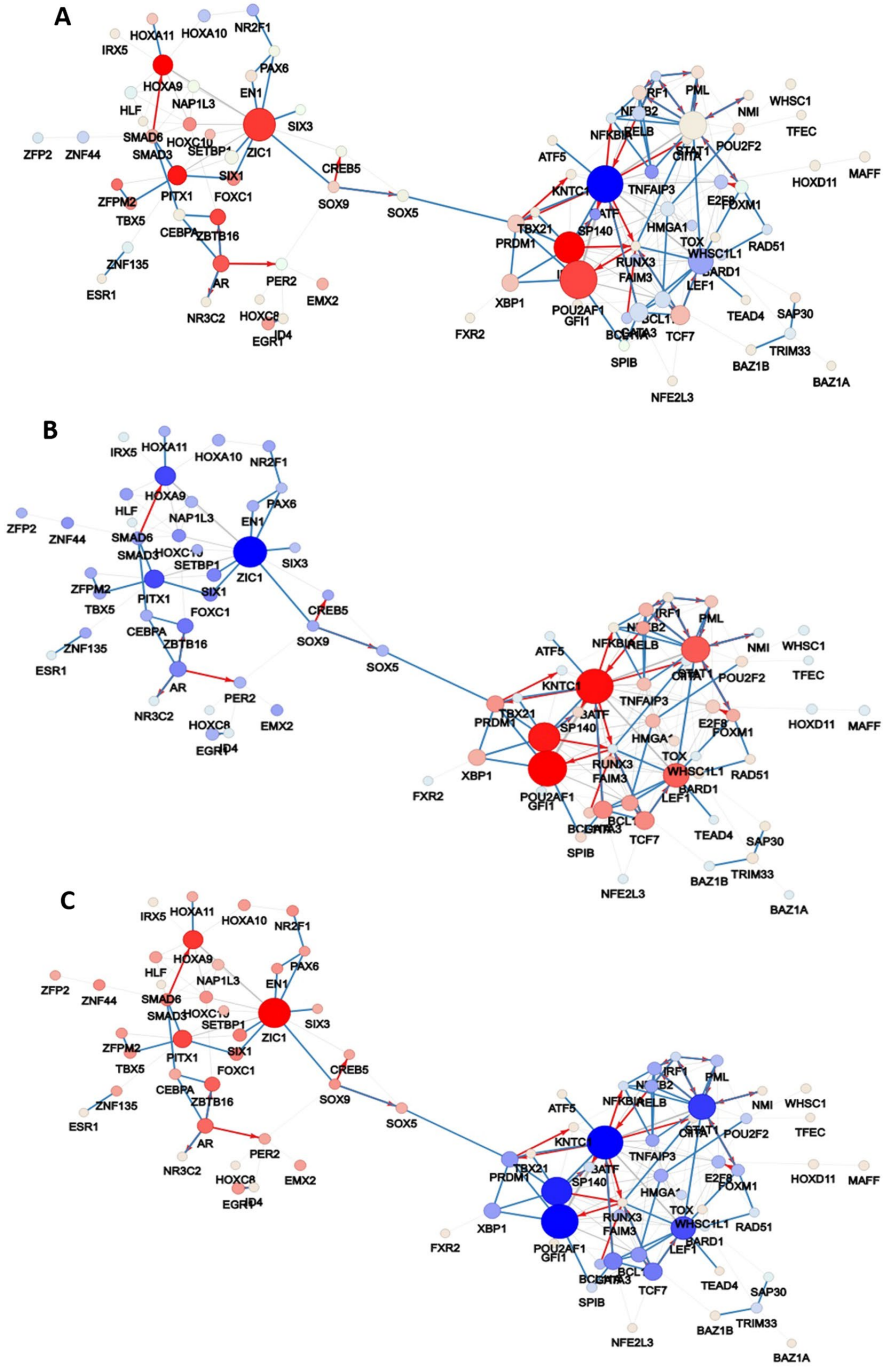
(**A**) The enriched network of the 126 TFs visualised in CoRegNet. The network was inferred using the DEG list of samples of synovial tissue between OA and RA patients. (**B**) Co-regulatory network showing the TF influence on the RA samples and (**C**) OA samples. The sphere size is proportional to the number of target genes of the TF/co-TF pairs. Lines between spheres represent the interaction between TFs/co-TFs pairs. The red colour indicates a strong TF influence and the blue colour indicates a weak TF influence.

We reconstructed a putative co-regulatory network using gene expression data from synovial tissue of RA patients using as control synovial tissue from OA patients, of three datasets: GSE55235, GSE55457 and GSE55584. We performed normalization and meta-analysis of the three combined datasets and obtained a list of 2865 differentially expressed genes (DEGs). Using the DEG list and the R/Bioconductor package CoRegNet^24^, we obtained a co-regulatory network. This network was then enriched with supporting regulatory evidence such as the TF binding site, ChIP-seq data, and protein interactions found in the CoRegNet embedded databases HIPPIE^27^, CHEA^28^, ENCODE^29^ and STRING^30^. The enriched network contained 126 TF and 1540 target genes linked together by 9716 TF-to-gene regulatory interactions. The TF-TF co-regulatory network is composed of 989 TFs pairs which are linked together by co-regulatory interactions (Fig. 2A). With CoRegNet we can identify the most influential TFs (Table 1) based on their influence score, and we can also retrieve information about the co-regulatory pairs in our network that share common targets (Table S1).

**Table 1:** Top 5 of the most influential TFs identified in samples of RA synovial tissue, using CoRegNet and microarray data.

Transcription factor	Influence in RA samples using CoRegNet
POU2AF1	457,54
BATF	451,96
IRF4	399,73
STAT1	370,34
LEF1	335,79

To identify which of the TFs have a strong influence in the RA and which in the OA synovium, we used the reference network to visualize the TF influences for the RA and OA samples separately (Fig. 2B,C). This way, we were able to identify the TFs that are active only in the RA synovium (Fig. 2B). POU2AF1 and BATF appear as the most influential TFs on the gene expression in synovial tissue of RA patients followed by IRF4, STAT1 and LEF1 (Table 1, Table S2). It is worth noting that the network consists of two distinct clusters connected by one TF, SOX5.

### Estimation of TFs influence in synovial fibroblasts using microarray gene expression

Next, we analyzed the dataset GSE107105 comprising seven RA FLS subpopulations and five OA FLS subpopulations, clustered according to their protein surface markers (CD34, THY1 and CDH11). For this dataset we calculated TF influence in two levels:

- disease level, analyzing all RA FLS samples against all OA FLS used as a control, and
- for each fibroblast subpopulation, comparing between similar subsets of RA and OA FLS, where possible.

For the analysis, and the TF influence estimation we used the whole expression matrix. Using the reference network, we calculated TF activities, and we were able to identify four out of the five TF considered as master regulators in RA synovial tissue data (Tables 2). NFKB2 was estimated as the fifth most influential TF for the RA samples. POU2AF1 was not identified, but POU2F2, its target, was found in the top 20 of most active TFs (Table S3).

**Table 2:** Top 5 of the most influential TFs identified in samples of RA FLS, using CoRegNet and microarray data.

Transcription factors	Influence in RA samples using CoRegNet
BATF	43,02
STAT1	38,18
LEF1	27,78
IRF4	26,25
NFKB2	25,28

Next, we analyzed the TF activities of the different subpopulations depending on the surface markers, separately. We analyzed data from seven RA and five OA FLS subpopulations (Table 3). We were not able to retrieve expression data for the C and G OA FLS. Corresponding co-regulatory networks for each FLS subpopulation are available in the Supplementary material (Figures S1, S2, S3, S4, S5). Comparing TF activities between the subpopulations revealed a dominant RA FLS TF activity profile where all five TF previously identified as master regulators in RA appear in the top 5 for most of the RA FLS types, with some slight variations. However, populations E and F exhibit an entirely different profile that is highly similar to the ones obtained for OA FLS. POU2AF1 is absent from all subpopulations of RA FLS. Concerning OA FLS it is worth noting that the dominant TFs in all subpopulations are TFs of the HOX family and SIX1 and 3.

**Table 3:** FLS subpopulations according to their surface proteins and the corresponding top 5 TFs identified in samples of OA and RA FLS (microarray data).

FLS subpopulations	CD34	THY11	CDH11	Top 5 TF in OA FLS	Top 5 TF in RA FLS
A	−	−	+	SIX3, HOXA9, HOXC10, PPARG, SIX1	**STAT1, BATF, IRF4**, NFKB2, **LEF1**
B	−	−	−	SIX3, HOXA9, FOXC1, SIX1, EN1	**BATF, IRF4, STAT1,** NFKB2, SP140
C	−	+	−	Data not available	**BATF**, PRDM1, NFKB2, **IRF4**, FOXM1
D	−	+	+	HOXC10, HOXA9, AR, SIX1, SIX3	**STAT1, BATF, LEF1**, NFKB2, PML
E	+	−	+	HOXC10, ZBTB16, HOXA9, HLF, EMX2	*ZNF135, ZBTB16, HOXA9, HLF, HOXC10*
F	+	+	+	HLF, ZBTB16, EMX2, HOXA9, HOXC10	*EMX2, HLF, ZBTB16, ZNF44, AR*
G	+	+	−	Data not available	**BATF**, GATA3, **LEF1**, TCF7, PRDM1

In bold are highlighted the 5 identified TFs and in italic the TFs that differ considerably in the respected subpopulation.

### Calculating TFs influence from scRNA-seq data of isolated fibroblasts

**Figure 3:**
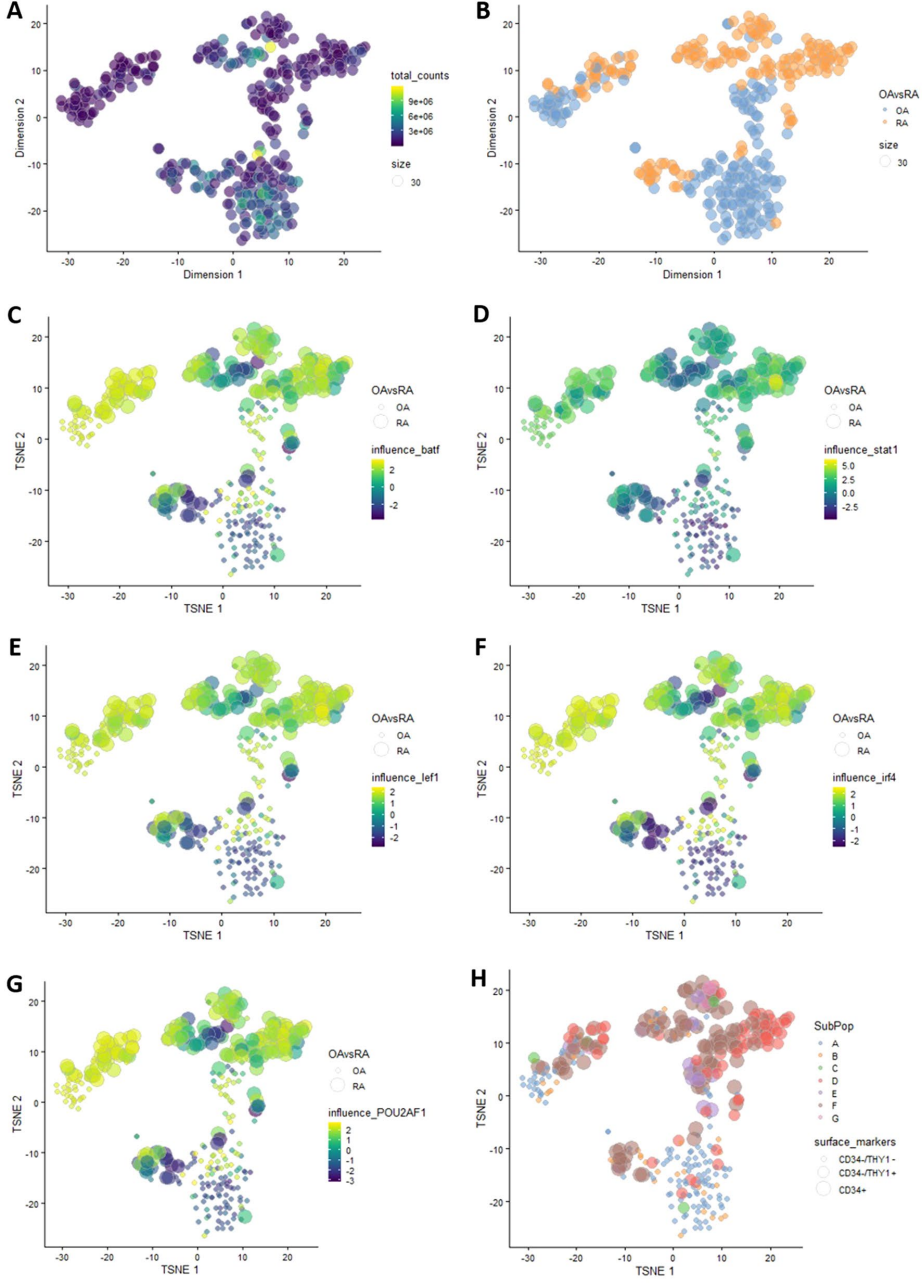
(**A**) tSNE clustering of scRNA-seq dataset, (**B**) Patients state projected on the tSNE output. (**C**) tSNE clustering showing the influence of BATF, (**D**) STAT1, (**E**) LEF1, (**F**) IRF4 and (**G**) POU2AF1 on both RA and OA samples. (**H**) Fibroblasts subpopulations projected on the tSNE clustering of scRNA-seq data. *OA* osteoarthritis, *RA* rheumatoid arthritis.

Finally, we analysed scRNA-seq data of isolated RA and OA FLS from two RA and two OA patients (GSE109449). First, we performed a dimensionality reduction using the tSNE algorithm^31^ (Fig. 3A). Then, we projected the patients’ disease state (OA/RA) on the plot, and we observed the clustering between OA and RA patients. As seen in Fig. 3B, the patients are separated into three clusters, based on their disease state. RA is the dominant case in one of the three clusters and OA in a second, however all three clusters have both cases. Subsequently, we calculated the TFs influence for the whole dataset using CoRegNet^24^.

The last step was to project on the tSNE clustering result, the TFs influence calculated using the whole dataset and not just DEGs. The aim was to see if at the single-cell level, the influence of the TFs identified as master regulators in RA synovium and RA FLS, would be considerably stronger in RA versus OA samples. Indeed, as shown in Figs. 3C–F, there is a concordance between the patients' disease state (OA/RA) and TFs influence. In essence, each of the identified TF has a strong influence on the gene expression of FLS from RA patients (coloured in green) while it has a weak influence on the gene expression of FLS from OA patients (coloured in purple). The five TFs previously identified as master regulators (STAT1, BATF, IRF4, LEF1 and POU2AF1) appear in the top 15 of the most influential TFs for RA FLS based on TF influence estimation using CoRegNet (Table S4). STAT1 and BATF are ranked as the second and the fifth most influential TFs, respectively.

Comparison of the top 5 of the most influential TFs in different FLS subpopulations revealed GATA3 as omnipresent in both RA and OA samples. We also observed the presence of STAT1 and BATF in most RA samples. Lastly, we obtained two very different TF activity profiles for the subpopulation C of the OA FLS and the subpopulation E for the RA FLS (Table 4). We did not observe any apparent clustering according to the surface markers independently of the disease state (Fig. 3F).

**Table 4:** FLS subpopulations according to their surface proteins and the corresponding top 5 TFs identified in samples of OA and RA FLS (scRNA-seq data).

FLS subpopulations	CD34	THY11	CDH11	Top 5 TF in OA FLS	Top 5 TF in RA FLS
A	−	−	+	SP140, GATA3, BCL11B, HMGA1, SPIB	GATA3, **STAT1**, SAP30, PRDM1, **BATF**
B	−	−	−	GATA3, BCL11B, TCF7, SPIB, HMGA1	GATA3, PML, **STAT1**, HMGA1, TNFAIP3
C	−	+	−	*TNFAIP3, RELB, NFKB2, NFKBIA, POU2F2*	GATA3, **STAT1**, HMGA1, SP140, **BATF**
D	−	+	+	PRDM1, GATA3, SAP30, IRF4, TOX	GATA3, **STAT1**, RELB, PML, BCL11A
E	+	−	+	GATA3, STAT1, FOXM1, LEF1, BATF	*PER2, SAP30, ZNF44, ZFP2, E2F8*
F	+	+	+	GATA3, FOXM1, LEF1, TOX, SAP30	GATA3, PML, **STAT1, BATF**, TOX
G	+	+	−	GATA3, STAT1, TCF7, HMGA1, BATF	GATA3, RELB, **BATF**, BCL11A, **STAT1**

In bold are highlighted the 5 identified TFs and in italic the TFs that differ considerably in the respected subpopulation.

### Estimation of transcriptional activities of the master regulators identified by CoRegNet, using ISMARA and DoRothEA

In order to see if the TFs identified as master regulators in the RA synovium and FLS samples using the TF influence and the reference network obtained from CoRegNet^24^ could be retrieved using an independent approach, we analyzed the datasets with two additional software (Table 5):

- ISMARA^21^, which estimates the TFs activities by identifying the promoters, quantifying their genome-wide expression and then fitting a linear model, and
- DoRothEA^20^ a framework to estimate TF activities from gene expression data and consensus TF-target binding networks.

**Table 5:** Results of ISMARA and DoRothEA analysis of the microarray and scRNA-seq datasets regarding the five master regulators identified by CoRegNet.

Transcription factor	Synovial tissue microarray ISMARA Z-value/ranking	RA FLS microarray ISMARA Z-value/ ranking	Synovial tissue microarray DoRothEA ranking	RA FLS microarray DoRothEA ranking	RA FLS scRNA-seq DoRothEA ranking
POU2AF1	Not identified	Not identified	Not identified	Not identified	Not identified
BATF	6.90/1	1.72/ 57	10	235	22
IRF4	2.76/96	2.69/11	11	6	12
STAT1	1.30/264	0.41/426	3	10	9
LEF1	3.05/74	1.18/134	115	213	75

#### Microarray data of synovial tissue samples

Using ISMARA, BATF was identified as the top TF with the highest Z-score. IRF4, STAT1 and LEF1 were also retrieved in various rankings. POU2AF1 was not identified but we were able to retrieve the target of POU2AF1, POU2F1 (Table S5). With DoRothEA, STAT1, BATF and IRF4 were found in the top 15 of the most active TFs in the RA synovium. POU2AF1 was not identified but its targets, POU2F1 and POU2F2 could be retrieved in the analysis in various rankings (Table S6).

#### Microarray data of FLS

Using ISMARA and DoRothEA, BATF, STAT1, IRF4 and LEF1 were retrieved in various rankings. Similarly to previous analyses, POU2AF1 was not identified, but its targets POU2F1 and POU2F2 were retrieved in the analysis in various rankings (Tables S7, S8).

#### scRNA-seq data of FLS

ISMARA analysis was not possible as the FASTQ files were not available. Analysis with DoRothEA identified STAT1, IRF4 and BATF in the top 25 (Table S9).

### Involvement of the master regulators in primary RA FLS phenotypic outcomes

To study the implication of the five identified master regulators to the major functional phenotypic outcomes of RA FLS, namely apoptosis, inflammation, proliferation and migration/invasion, we retrieved hallmark molecular signatures from MSig database^26^. We compared the signatures with the differentially expressed genes in the scRNA-seq data set (GSE109449). We kept only the genes having a p-value lower than 0.05 and a log fold change (logFC) higher than 1.5 in absolute value (Tables S10–S13).

**Figure 4:**
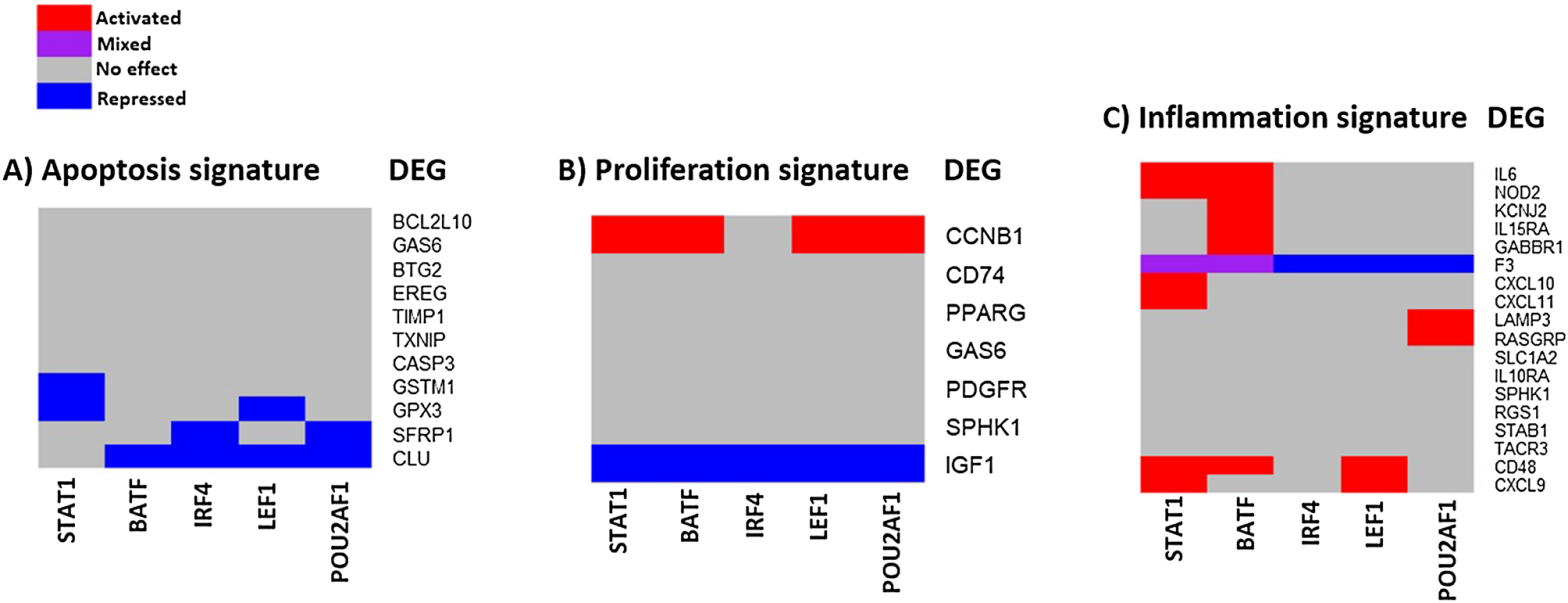
DEGs in the scRNA-seq dataset belonging to hallmark signatures, (**A**) Apoptosis, (**B**) Proliferation, (**C**) Inflammation, as compared to gene sets derived from MSig database. The heatmap indicates regulation (or absence of) by the five identified master regulators, STAT1, BATF, IRF4, LEF1 and POU2AF1.

Next, we searched to see which of the genes identified as contributing to hallmark signatures were under the control of the five TFs characterized as master regulators (Fig. 4). We identified 12 DEGs that belong to the pro-apoptotic signature, 13 DEGs that belong to the proliferation signature and 18 DEGs that belong to the inflammatory signature. Concerning the migration markers we identified THBS1 and also MMP-14, an enzyme principally involved in FLS invasion of collagenous structures, including articular cartilage^32^ when using slightly less stringent criteria (MMP14 logFC: 1,38 and p-value: 5,2891E−06). However, neither THBS1 nor MMP14 are under the control of the five master regulators.

The master regulators can cause downregulation of four DEGs belonging to the apoptotic signature, namely CLU, SFRP1, GPX3 and GSTM1 (Fig. 4A), activate the expression of one proliferation marker, CCNB1 and downregulate another, IGF1 (Fig. 4B), and activate eleven genes that contribute to inflammation including IL6, a major inflammatory cytokine in RA (Fig. 4C).

## Discussion

Synovial fibroblasts are the main stromal cells in the synovial tissue of the joints. In healthy joints, they are found in the synovial lining and sublining layer (intima and subintima), forming a layer with a thickness of one to two cells, intercalated with tissue-resident macrophages^10,33^. In RA, synovial fibroblasts start to form thicker layers (15–20 cells thick) mainly due to a higher rate of proliferation and also due to a characteristic apoptosis-resistant phenotype. Immune cells infiltrate the sublining layer of the synovium, and the lining layer thickens, even more, resulting in the hallmark pannus formation. Activated synovial fibroblasts produce a variety of ECM remodelling components such as MMPs, and also cytokines and chemokines, contributing actively to cell recruitment and infiltration of the joint, and sustained and prolonged inflammation of the joint. Activated synovial fibroblasts can also invade and destroy the adjacent cartilage, contributing to cartilage damage and subsequent bone erosion, two major debilitating symptoms of RA. In summary, RA FLS differ from healthy synovial fibroblasts or even, OA FLS in the four following phenotypic traits: (a) resistance to apoptosis, (b) migratory and invasive capacities, (c) inflammatory responses, (d) higher proliferation rate.

In this study, we tried to characterize transcription factors that could potentially control and regulate these distinct phenotypic traits of RA FLS based on the assumption that these traits are the result of a distinct TF activity profile in comparison to control.

While in synovial tissue, other cells, like macrophages, leukocytes, plasma cells and mast cells are present, RA FLS is the dominant cell population^34^. Transcriptomic data of synovial tissue were used to infer a global, reference network of tissue-specific transcriptional activity. As a control, we used synovial tissue from OA. OA is a degenerative, non-autoimmune and non-inflammatory disease of the joints that also causes cartilage damage and bone erosion^35^. Inflammatory mediators observed in OA joints are thought to be the downstream effectors of the pathogenesis of the disease. Even though it’s called non-inflammatory arthritis, OA can still result in some inflammation of the joints. The difference is that this inflammation probably results from wear and tear, as a secondary effect of the disease and not as a characteristic feature. Moreover, OA is a degenerative disease, while RA involves autoimmunity. While it is hard to distinguish between a characteristic feature and a secondary effect, the fact that inflammation in RA stems from the activated autoimmune pathways, allows us to hypothesize that there should exist different mechanisms that drive the onset, progression and pathogenesis of the two diseases. For the analysis, we used CoRegNet^24^, a tool for network inference based on TF influence estimation. The tool CoRegNet has successfully been used to identify driver regulators for bladder cancer^36^, master regulators in fatty liver disease^37^ and hepatocellular carcinoma^38^ and sex-specific gene regulation in drosophila^39^, among other studies. Two additional software was used for calculating TF activities of the samples, for seeing if we could retrieve the TFs already identified with CoRegNet, independently of the reference network.

Two opposite TF influence profiles were obtained for the OA patients and the RA patients using the global network inferred from the synovial tissue gene expression. This result corresponds to the fact that we used only the differentially expressed genes between RA and OA patients to perform the network inference so that only autoimmune changes specific to RA are revealed.

Five TFs having a strong influence on RA gene expression were detected. BATF, a member of the AP-1 family transcription factor that controls the differentiation of lineage-specific cells in the immune system. BATF mediates the differentiation of T-helper 17 cells (Th17), follicular T-helper cells (TfH), CD8^+^ dendritic cells and class-switch recombination (CSR) in B-cells^40^. In chondrocytes, overexpression of BATF leads to increased mRNA and protein levels of the matrix-degrading enzymes MMP3, MMP13 and ADAMTS5, which play crucial roles in OA cartilage destruction^41^. BATF overexpression in joint tissues causes synovial inflammation suggesting that BATF could contribute to inflammatory arthritis. BATF has been shown to regulate synovitis, synovial hyperplasia, angiogenesis, cartilage destruction, and bone erosion in the joint tissues^42^.

STAT1, a member of the STAT protein family that can be activated by various ligands including interferon-alpha, interferon-gamma, EGF, PDGF and IL6. High expression of STAT1 is intrinsic to RA fibroblast-like synoviocytes in the intimal lining layer, whereas activation of the pathway by phosphorylation is an active process^43^. Raised levels of total STAT1 protein and both its activated tyrosine and serine phosphorylated forms were also seen in RA synovium as compared with controls. STAT1 is predominantly abundant in T and B lymphocytes in focal inflammatory infiltrates and in fibroblast-like synoviocytes in the intimal lining layer^44^.

POU2AF1 (POU Class 2 Homeobox Associating Factor 1) is a transcriptional coactivator that associates specifically with either POU2F1 or POU2F2. It boosts the POU2F1 mediated promoter activity and to a lesser extent, that of POU2F2. POU2AF1 was identified when comparing arthritis versus healthy or ACPA + individuals, suggesting that the downregulation of such genes starts after the onset of symptoms in RA patients. Also, a significant correlation was identified for POU2AF1 and disease progression with a downward trend for those with established RA^19^.

IRF4 is a TF is required for proper maturation and differentiation of immune cells. It is expressed in cells relevant for IFN signature in autoimmunity such as dendritic cells, monocytes/macrophages, granulocytes and B-cells. Moreover, IRF4 loci are associated with genetic susceptibility to systemic autoimmune diseases^45^. IRF4 has been related to NFkB pathway and can also interact with MyD88, an adaptor protein crucial for the activation of IRGs^45^.

The last identified TF is LEF1 for Lymphoid enhancer-binding factor-1 (LEF1), a 48-kD nuclear protein that is expressed in pre-B and T cells. LEF1 participates in the Wnt signalling pathway which regulates fibronectin and metalloproteinase expression in RA FLS^46^ .

An interesting observation concerning the reference network is that it consists of two distinct clusters united by a single TF, SOX5. While SOX5 did not appear to have a strong influence regarding the target genes, it can be considered as a structural hub of the reference network. SOX5 was reported recently to contribute to the migration and invasion of FLS in collagen-induced arthritis^47^. SOX5 has also been suggested as an important regulator of IL-6-induced RANKL expression in RA FLS^48^.

The five TFs identified as master regulators can be considered disease-related for RA, as for the inference of the reference network we used the DEGs between samples of synovial tissue from RA and OA patients.

To verify that these TFs also play a role in RA FLS and they were not the result of the contribution of other cells present in the synovial tissue samples, the second step of our study involved the analysis of transcriptomic data from RA and OA FLS subpopulations. These datasets are related to Mizoguchi et al. study^49^ where seven fibroblast subsets with distinct surface protein (CD34, THY1 and CDH11) phenotypes were isolated. Using the reference network obtained from the synovial tissue as a basis, we calculated the TF influence of the FLS transcriptome. We performed this step twice, one for comparing TF profiles in RA FLS versus OA FLS, and subsequently for comparing TF activities between subpopulations of RA and OA FLS sharing the same surface markers.

The first part of the analysis revealed that four out of the five identified TFs as master regulators for the RA synovium were also characteristic for the RA FLS samples. POU2AF1 was not retrieved in the microarray dataset for FLS. Instead, the TF NFKB1 appeared in the top 5. Analysis comparing RA and OA FLS according to their surface markers revealed two RA FLS subpopulations, E and F respectively, with a significantly different TF activity profile in comparison to the other RA FLS subpopulations.

The analysis of the scRNA seq data revealed STAT1, BATF, IRF4, LEF1 and POU2AF1 in the top 15 of the most influential TFs for RA FLS, with STAT1 and BATF ranking as the second and the fifth most influential TF, respectively. Regarding the subpopulation-specific TF activity profiles, STAT1 and BATF were dominant in RA samples. The transcription profiles of the subpopulation C of the OA FLS and the subpopulation E of the RA FLS were very different in comparison to the other subpopulations. Lastly, we did not observe any apparent clustering according to surface markers.

Transcription factor activity of the master regulators was also estimated with two additional software, ISMARA and DoRothEA. Unlike these tools, CoRegNet identifies cooperative regulators of genes. It considers, rather than the expression of regulators, their influence to detect co-regulators sharing a certain number of common targets. The results are not directly comparable since the three software use different algorithms to assess TFs activity, however most of the TFs identified with CoRegNet as having a strong influence in RA FLS samples were also retrieved from the ISMARA and DoRothEA analysis, confirming in an independent manner that these TFs do play an active role in RA FLS.

Analysis of the DEGs in the scRNA-seq data revealed genes implicated in hallmark molecular signatures related to major functional phenotypic outcomes of RA FLS, namely apoptosis, inflammation, proliferation and migration/invasion. Further analysis using the co-regulatory network obtained with CoRegNet highlighted genes under the control of the master regulators that are involved in apoptosis (suppressing pro-apoptotic markers), proliferation (suppressing proliferation markers) and inflammation (activating inflammatory markers). Very few characteristic migration markers were differentially expressed and none under the control of the master regulators identified.

In conclusion, this study contributes to a better understanding of RA FLS regulation by identifying disease- and cell-specific master regulators, some highlighted for the first time as playing a pivotal role in fibroblasts. In an effort to limit misleading results and misinterpretations, statistical tests were performed—where feasible—to ensure homogeneity of the datasets used for sex and age (see Methods section and supplementary Tables 14–16). It should be noted, though, that besides age and sex, medical treatments and disease duration may potentially affect the TF levels in the patients. However, there are technical difficulties in accessing this type of data (often missing from original studies, not published along the datasets in public repositories, not available for all selected datasets, supplementary data not accessible) that do not allow for further adjustments using appropriate statistical tests. To ensure biological relevance of our results we have used, besides our main methodology, two independent software for cross validation, and finally we have cross checked our findings with experimental results in peer-reviewed RA studies. Experimental validation of the TFs identified as putative master regulators using in vitro settings and additional analyses are required to understand how different TF activity profiles coordinate gene expression in RA synovium and RA FLS subsets, determining disease and cell-specific phenotypic traits, cellular destiny and disease severity.

## Methods

### Data description and analysis for the inference of the co-regulatory reference network

For the inference of the co-regulatory reference network, we used three genome-wide transcriptomic datasets from the Affymetrix HG- U133 A/B array comprising a total of 79 samples of synovial tissue, including 20 Controls (C), 26 OA and 33 RA patients. The first dataset (Berlin dataset, GSE55235) includes 30 samples: 10 RA, 10 OA and 10 C. The second dataset (Jena dataset, GSE55457) includes 33 samples: 10 C, 13 RA and 10 OA. The third dataset (Leipzig, GSE55584) includes 16 samples: 6 OA and 10 RA. We used R version 3.5.3 to perform the exploratory data analysis and the differential expression analysis, with an eight-core processor, with 32Go of RAM running on Windows 10 (64 bits) and the Affy package version 1.62^50^. We normalised the genes expression using the *mas5* function from the Affy package. Then we deleted the weakly expressed genes with the function *mas5calls* which performs the Wilcoxon signed rank-based gene expression presence/absence detection algorithm. Next, we performed differential expression analysis to identify genes that are differentially expressed between RA and OA patients using as a significance threshold an adjusted p-value (FDR) equal to 0,05. As the number of controls was lower than the number of OA samples, we decided to use OA samples as reference. OA remains a reasonable control for RA since OA is a non-inflammatory, non-autoimmune disease. We annotated the lists of DEGs using the file provided by Affymetrix. The annotation allowed us to retrieve the GenBank accession number and Gene Symbol for each probe. To address the problems of several probes corresponding to the same gene and of several genes having several symbols (aliases) we averaged the expression of each gene by calculating the mean of all the gene’s probes. After that, for each gene, we kept all the possible aliases. The differential expressed gene list contained 2865 gene symbols, and it was used for the inference of the co-regulatory network.

### Inference of the co-regulatory reference network

CoRegNet is a Bioconductor R-package. Based on a transcriptomic dataset, it can be used to reconstruct a co-regulatory network and integrate regulation evidence. The package uses the hLICORN algorithm^51^, which identifies cooperative regulators of genes. One key breakthrough in the exploration of networks was to consider, rather than the expression of regulators, their influence by evaluating the expression of target genes to detect master regulators. We used the CoRegNet package^24^ version 1.20.0 to infer the network. We gave it as input the annotated expression matrix with the gene symbol as row names and the samples (OA/RA) as columns. Then, we run in parallel on 48 cores, the function *hLICORN* to obtain the co-regulatory network. Regulatory and cooperative evidence were used for gene regulatory network and co-regulatory network enrichment.

### Calculating TF activities with ISMARA and DoRothEA

To validate the results and see if the TFs identified as master regulators in the reference network with CoRegNet could be retrieved with a different approach, we employed in our analysis two additional software which calculates TFs activity: ISMARA^21^ and DoRothEA^20^. ISMARA is an online tool that models genome-wide expression or ChIP-seq data, in terms of computationally predicted regulatory sites for transcription factors. The input required for running ISMARA is either expression data (microarray CEL files) or ChIP-seq data to identify the key transcription factors driving the observed expression. Results include, for each regulatory motif, inferred activities across the input samples, predicted genome-wide targets, enriched pathways and functional classes of genes, and direct interactions between regulators. This tool uses a linear model and performs a Bayesian inference of the motif activities to obtain both best-fit activities and error-bars on the activities. The second tool, DoRothEA, is an R package containing a list of transcription factors and their transcriptional targets. The target genes are organized into regulons coming from different types of evidence like literature curated resources and interactions inferred directly from gene expression. Based on the expression of each regulon, the tool estimates the activity of its corresponding TF.

### TFs influence generation using fibroblasts gene expression microarray dataset

We used microarray data of freshly isolated synovial fibroblast subsets in patients with RA and OA to refine the previously obtained results. The dataset GSE107105 includes 34 samples from three OA and three RA donors. First, we generated TF influence profiles for RA and OA patients and then, for each fibroblast subpopulation. Next, we used the co-regulatory network inferred at the previous step and the influence of the co-regulators to detect possible variations.

### TFs influence generation using scRNA-seq dataset

We used scRNA-seq data we got from the GEO database (GSE109449) collected from freshly isolated synovial fibroblasts of 2 RA patients and 2 OA patients. The fibroblasts were gated by protein surface markers: CD34, THY1 and CDH11 (126 samples for A, 41 samples for B, four samples for C, 65 samples for D, 13 samples for E, 92 samples for F and four samples for G subpopulation, for correspondence, see Tables 3 and 4).

The first step of the analysis was to preprocess the data. We used Single Cell Experiment R package^52^. It is specifically designed for storing data from sc experiments. Then we filtered low abundance genes because most of them are non-informative and are not representative of the biological variance of the data. We only kept genes having an average expression level higher than one. We also filtered genes that are in a small number of cells by keeping only the ones expressed in at least five cells. A complementary way to filter genes is to keep only protein-coding genes. scRNASeq profiles not only protein-coding genes but also long non-coding RNA and pseudogenes. These genes are often much noisier than protein-coding and might be interesting to remove them. To do that, we annotated the expression matrix using Biomart R package and kept the genes having “protein-coding” as biotype. After that, we performed quality control using isOutlier function from Scater R package to filter low-quality cells and low-quality features.

The following step of our analysis was to normalize the data using a size factor approach. Once the quality control finished, we identified the highly variable genes (HVG). To do that, we calculated the variance and the mean expression for each gene. Then, we performed a dimensionality reduction using the tSNE algorithm^31^. In addition to the tSNE clustering, we used hierarchical clustering and SC3 clustering, an unsupervised clustering method for scRNA-seq data^53^. Finally, we used scRNA-seq data and CoRegNet to calculate the influence of the transcription factors present in this dataset on the gene expression of FLS from RA and OA patients. To validate the results we got from this dataset, we applied ISMARA and DoRothEA for calculating the TFs activity.

### Proliferation, apoptosis, migration/ invasion and inflammation markers identification :

First, we retrieved hallmark gene sets from MSig database^26^. Two hundred genes were retrieved for the inflammatory pathway, 171 plus 3 fibroblast specific genes for the apoptosis pathway, 40 fibroblast specific genes for the migration/invasion pathway and 85 fibroblast specific genes for the proliferation pathway.The expression profiles of these markers come from: Genotype-Tissue expression (GTEx) project data^54^, Human Tissue compendium data^55^, The NCI-60 Human Tumor Cell Lines Screen data^56^ and the Global Cancer Map from Broad Institute^57^. The fibroblast specific gene sets were contributed by the Gene Ontology consortium^58^.

We performed a differential expression analysis using the Limma package^59^ to the scRNA-seq data (GSE109449) to see which of the corresponding markers have differential expression between RA and OA patients. We kept only the genes having a p-value lower than 0.05 and a log fold change higher than 1.5 in absolute value.Then, for the apoptosis pathway, we kept all the overexpressed antiapoptotic markers and all the underexpressed proapoptotic ones. For the inflammation, proliferation and migration, we only kept the overexpressed markers having a positive regulation and the underexpressed markers having a negative regulation on these pathways. Finally, we searched to see which of these genes are under the control of the five identified master regulators based on the co-regulatory network inferred by CoRegNet.

### Correction for the age and sex of the patients

The distribution of the sex between RA and OA patients is similar in the synovial tissue samples (p-value Fisher test = 0.52) and in the fibroblasts microarray samples (p-value Fisher test = 1). In the fibroblast single cell samples, all individuals were females. Therefore, we did not adjust for sex.

Concerning the age, we did not have information for each individual in the synovial tissue samples (only the mean age at onset for RA and OA). Therefore, we did not adjust for age.

A pairwise comparison was also performed for the sex and age between the OA patients of the 3 datasets and the RA patients of the 3 datasets (for age, the synovial tissue samples were not considered as explained above). The sex and age were homogeneously distributed between OA patients (p-value Fisher test > 0.45 for sex and p-value Wilcoxon test = 0.8 for age) and RA patients (p-value Fisher test = 1 for sex and p-value Wilcoxon test = 0.8 for age) of the different datasets. Details for datasets’ characteristics, statistical tests and corresponding p-values can be found in Supplementary Tables 14–16.

## Supplementary information

Supplementary Information

## References

[1] McInnes IB, Schett G (2011). The pathogenesis of rheumatoid arthritis. N. Engl. J. Med..

[2] Bartok B, Firestein GS (2010). Fibroblast-like synoviocytes: Key effector cells in rheumatoid arthritis. Immunol. Rev..

[3] Guo Q (2018). Rheumatoid arthritis: Pathological mechanisms and modern pharmacologic therapies. Bone Res..

[4] Yap H-Y (2018). Pathogenic role of immune cells in rheumatoid arthritis: Implications in clinical treatment and biomarker development. Cells.

[5] Jones DS (2017). Profiling drugs for rheumatoid arthritis that inhibit synovial fibroblast activation. Nat. Chem. Biol..

[6] Siebert S, Tsoukas A, Robertson J, McInnes I (2015). Cytokines as therapeutic targets in rheumatoid arthritis and other inflammatory diseases. Pharmacol. Rev..

[7] Firestein GS (2003). Evolving concepts of rheumatoid arthritis. Nature.

[8] Li F (2019). Nomenclature clarification: Synovial fibroblasts and synovial mesenchymal stem cells. Stem Cell Res. Ther..

[9] Kung M, Markantonis J, Nelson S, Campbell P (2015). The synovial lining and synovial fluid properties after joint arthroplasty. Lubricants.

[10] Ouboussad L, Burska AN, Melville A, Buch MH (2019). Synovial tissue heterogeneity in rheumatoid arthritis and changes with biologic and targeted synthetic therapies to inform stratified therapy. Front. Med..

[11] Bottini N, Firestein GS (2013). Duality of fibroblast-like synoviocytes in RA: Passive responders and imprinted aggressors. Nat. Rev. Rheumatol..

[12] Li H, Wan A (2013). Apoptosis of rheumatoid arthritis fibroblast-like synoviocytes: Possible roles of nitric oxide and the thioredoxin 1. Mediators Inflamm..

[13] Tang MW (2018). Class 3 semaphorins modulate the invasive capacity of rheumatoid arthritis fibroblast-like synoviocytes. Rheumatology.

[14] Carro MS (2009). The transcriptional network for mesenchymal transformation of brain tumours. Nature.

[15] Osmanbeyoglu HU, Toska E, Chan C, Baselga J, Leslie CS (2017). Pancancer modelling predicts the context-specific impact of somatic mutations on transcriptional programs. Nat. Commun..

[16] Schacht T, Oswald M, Eils R, Eichmüller SB, König R (2014). Estimating the activity of transcription factors by the effect on their target genes. Bioinformatics.

[17] Alvarez MJ (2016). Functional characterization of somatic mutations in cancer using network-based inference of protein activity. Nat. Genet..

[18] Falco MM, Bleda M, Carbonell-Caballero J, Dopazo J (2016). The pan-cancer pathological regulatory landscape. Sci. Rep..

[19] Romo-García MF (2019). Evaluation of SUMO1 and POU2AF1 in whole blood from rheumatoid arthritis patients and at risk relatives. Int. J. Immunogenet..

[20] Garcia-Alonso LM (2017). Transcription factor activities enhance markers of drug sensitivity in cancer. Cancer Res..

[21] Pachkov M (2017). ISMARA: Completely automated inference of gene regulatory networks from high-throughput data. PeerJ.

[22] Cholley P-E (2018). Modeling gene-regulatory networks to describe cell fate transitions and predict master regulators. NPJ Syst. Biol. Appl..

[23] Janky R (2014). iRegulon: From a gene list to a gene regulatory network using large motif and track collections. PLoS Comput. Biol..

[24] Nicolle R, Radvanyi F, Elati M (2015). CoRegNet: Reconstruction and integrated analysis of co-regulatory networks: Fig. 1. Bioinformatics.

[25] van de Peppel J (2017). Identification of three early phases of cell-fate determination during osteogenic and adipogenic differentiation by transcription factor dynamics. Stem Cell Rep..

[26] Liberzon A (2011). Molecular signatures database (MSigDB) 3.0. Bioinformatics.

[27] Alanis-Lobato G, Andrade-Navarro MA, Schaefer MH (2017). HIPPIE v20: enhancing meaningfulness and reliability of protein–protein interaction networks. Nucleic Acids Res..

[28] Lachmann A (2010). ChEA: Transcription factor regulation inferred from integrating genome-wide ChIP-X experiments. Bioinformatics.

[29] Davis CA (2018). The encyclopedia of DNA elements (ENCODE): Data portal update. Nucleic Acids Res..

[30] Szklarczyk D (2017). The STRING database in 2017: Quality-controlled protein–protein association networks, made broadly accessible. Nucleic Acids Res..

[31] van der Maaten L, Hinton G (2008). Visualizing data using t-SNE. J. Mach. Learn. Res..

[32] Asif Amin M, Fox DA, Ruth JH (2017). Synovial cellular and molecular markers in rheumatoid arthritis. Semin. Immunopathol..

[33] Ospelt C (2017). Review: Synovial fibroblasts in 2017. RMD Open.

[34] Bromley M, Woolley DE (1984). Histopathology of the rheumatoid lesion. Arthritis Rheum..

[35] Pap T, Korb-Pap A (2015). Cartilage damage in osteoarthritis and rheumatoid arthritis—two unequal siblings. Nat. Rev. Rheumatol..

[36] Nicolle, R. Regulatory networks driving bladder cancer. (Evry-Val d’Essonne, 2015).

[37] Lou Y (2017). Potential regulators driving the transition in nonalcoholic fatty liver disease: A stage-based view. Cell. Physiol. Biochem..

[38] Li Z (2019). Discovering master regulators in hepatocellular carcinoma: one novel MR, SEC14L2 inhibits cancer cells. Aging.

[39] Wang Y (2018). Reprogramming of regulatory network using expression uncovers sex-specific gene regulation in Drosophila. Nat. Commun..

[40] BATF protein (human)-STRING interaction network. https://string-db.org/network/9606.ENSP00000286639.

[41] BATF-Basic leucine zipper transcriptional factor ATF-like - Homo sapiens (Human) - BATF gene & protein. https://www.uniprot.org/uniprot/Q16520.

[42] Park S-H (2018). BATF regulates collagen-induced arthritis by regulating T helper cell differentiation. Arthritis Res. Ther..

[43] Malemud CJ (2018). The role of the JAK/STAT signal pathway in rheumatoid arthritis. Therap. Adv. Musculoskelet. Dis..

[44] Kasperkovitz P (2004). Activation of the STAT1 pathway in rheumatoid arthritis. Ann. Rheum. Dis..

[45] Rodríguez-Carrio J (2018). IRF4 and IRGs delineate clinically relevant gene expression signatures in systemic lupus erythematosus and rheumatoid arthritis. Front. Immunol..

[46] Sen M (2002). Regulation of fibronectin and metalloproteinase expression by Wnt signaling in rheumatoid arthritis synoviocytes. Arthritis Rheum..

[47] Shi Y (2018). Transcription factor SOX5 promotes the migration and invasion of fibroblast-like synoviocytes in part by regulating MMP-9 expression in collagen-induced arthritis. Front. Immunol..

[48] Feng X (2016). Modulation of IL-6 induced RANKL expression in arthritic synovium by a transcription factor SOX5. Sci. Rep..

[49] Mizoguchi F (2018). Functionally distinct disease-associated fibroblast subsets in rheumatoid arthritis. Nat. Commun..

[50] Gautier, L. *et al.* Affy—analysis of Affymetrix GeneChip data at the probe level. *Bioinformatics* **20**, 307–315 (2004). 10.1093/bioinformatics/btg405 14960456

[51] Chebil I, Nicolle R, Santini G, Rouveirol C, Elati M (2014). Hybrid method inference for the construction of cooperative regulatory network in human. IEEE Trans. Nanobiosci..

[52] Lun, A. & Risso, D. SingleCellExperiment: S4 Classes for Single Cell Data. R package version 1.10.1. (2020).

[53] Kiselev VY (2017). SC3: Consensus clustering of single-cell RNA-seq data. Nat. Methods.

[54] BBRB. https://biospecimens.cancer.gov/resources/sops/gtex.asp.

[55] Hsiao LL (2001). A compendium of gene expression in normal human tissues. Physiol. Genom..

[56] Shoemaker RH (2006). The NCI60 human tumour cell line anticancer drug screen. Nat. Rev. Cancer.

[57] Cancer Dependency Map. *Broad Institute*. https://www.broadinstitute.org/cancer/cancer-dependency-map (2016).

[58] Gene Ontology Consortium (2015). Gene ontology consortium: Going forward. Nucleic Acids Res..

[59] Ritchie, M. E. *et al.* Limma powers differential expression analyses for RNA-sequencing and microarray studies. *Nucleic Acids Res.* **43**, e47 (2015). 10.1093/nar/gkv007 PMC4402510 25605792

