# Supplementary material for: Identification of putative master regulators in rheumatoid arthritis synovial fibroblasts using gene expression data and network inference
Source: Sci Rep. 2020 Oct 1;10:16236. doi: 10.1038/s41598-020-73147-4 (PMC7529794; doi:10.1038/s41598-020-73147-4)
Supplement: Supplementary file 1 — Supplementary Information [file 41598_2020_73147_MOESM1_ESM.docx]

**Identification of putative master regulators in rheumatoid arthritis synovial fibroblasts using gene expression data and network inference.**

**^1^Naouel Zerrouk, ^1^Quentin Miagoux, ^2^Aurelien Dispot, ^2^Mohamed Elati, ^1^Anna Niarakis***

1GenHotel, Univ. Évry, Université Paris-Saclay, 91025, Genopole, Évry, France

2Univ. Lille, CNRS, Inserm, CHU Lille, Centre Oscar Lambret, UMR9020 – UMR1277 - Canther – Cancer Heterogeneity, Plasticity and Resistance to Therapies, F-59000 Lille, France

*corresponding author

Université d’Evry Val d' Essonne, 2, rue Gaston Crémieux 91057 EVRY-GENOPOLE cedex

[anna.niaraki@univ-evry.fr](mailto:anna.niaraki@univ-evry.fr)

ORCID: 0000-0002-9687-7426

**Supplementary material**

**Table S1.** Top 20 co-regulators (regulator pairs) in the reference network. The co-regulators were calculated by coregulators function in CoRegNet package and sorted by adjusted P value. Fisher test determines the specificity of the shared targets between two regulators. The default p-value adjustment uses FDR correction to extract significant pairs of co-regulators.

| **Reg. 1** | **Reg. 2** | **Fisher Test** | **adjusted P value** |
| --- | --- | --- | --- |
| IRF4 | POU2AF1 | 9,554E-257 | 9,4489E-254 |
| IRF4 | XBP1 | 1,29581E-96 | 6,40777E-94 |
| POU2AF1 | XBP1 | 4,93381E-68 | 1,62651E-65 |
| LEF1 | TCF7 | 2,08905E-48 | 5,16519E-46 |
| NFKB2 | RELB | 9,1803E-44 | 1,81586E-41 |
| BCL11B | TCF7 | 6,84076E-39 | 1,12759E-36 |
| BCL11B | LEF1 | 1,31778E-38 | 1,86184E-36 |
| POU2AF1 | PRDM1 | 5,67885E-38 | 7,02048E-36 |
| HOXA9 | HOXC10 | 2,51529E-36 | 2,76402E-34 |
| GATA3 | TCF7 | 3,63598E-36 | 3,59598E-34 |
| BATF | TNFAIP3 | 1,51708E-35 | 1,36399E-33 |
| BCL11B | POU2AF1 | 2,0518E-32 | 1,69102E-30 |
| BCL11B | FAIM3 | 2,19159E-30 | 1,66729E-28 |
| NFKB2 | STAT1 | 3,0621E-29 | 2,16315E-27 |
| HOXA11 | HOXA9 | 6,63262E-27 | 4,37311E-25 |
| LEF1 | STAT1 | 1,90661E-25 | 1,1092E-23 |
| BATF | GATA3 | 1,82797E-25 | 1,1092E-23 |
| HOXA9 | ZIC1 | 5,46983E-25 | 3,00537E-23 |
| BCL11B | GATA3 | 1,1204E-24 | 5,83199E-23 |
| BATF | PRDM1 | 2,14077E-24 | 1,05861E-22 |

**Table S2 :** Top 20 of the most influential TFs in CoRegNet using microarray data of synovial tissue from RA and OA patients

| ID | influence in OA | influence in RA |
| --- | --- | --- |
| **POU2AF1** | -458,71 | 457,54 |
| **BATF** | -470,73 | 451,96 |
| **IRF4** | -400,17 | 399,73 |
| **STAT1** | -380,17 | 370,34 |
| **LEF1** | -352,68 | 335,79 |
| TCF7 | -287,27 | 271,64 |
| GATA3 | -271,45 | 265,72 |
| BCL11B | -236,92 | 224,21 |
| PRDM1 | -231,69 | 220,90 |
| XBP1 | -216,50 | 210,38 |
| NFKB2 | -195,23 | 203,13 |
| RELB | -195,64 | 201,84 |
| HMGA1 | -195,68 | 189,14 |
| TNFAIP3 | -188,94 | 179,29 |
| FOXM1 | -167,99 | 177,94 |
| PML | -147,18 | 156,83 |
| E2F8 | -172,58 | 153,07 |
| FAIM3 | -170,06 | 146,24 |
| BCL11A | -169,32 | 144,57 |

**Table S3** : TOP 20 of the most influential TFs in CoRegNet using microarray of isolated fibroblasts.

| ID | influence in OA | influence in RA |
| --- | --- | --- |
| **BATF** | -56,13 | 43,02 |
| **STAT1** | -47,46 | 38,18 |
| **LEF1** | -36,09 | 27,78 |
| **IRF4** | -33,86 | 26,25 |
| NFKB2 | -22,23 | 25,28 |
| PRDM1 | -30,33 | 24,18 |
| PML | -21,35 | 21,26 |
| GATA3 | -29,25 | 20,06 |
| TCF7 | -30,92 | 19,78 |
| BCL11B | -18,45 | 15,94 |
| RELB | -21,30 | 15,87 |
| BCL11A | -15,97 | 15,49 |
| XBP1 | -21,95 | 15,26 |
| TNFAIP3 | -19,21 | 14,87 |
| FOXM1 | -18,25 | 13,19 |
| NFKBIA | -12,71 | 12,89 |
| HMGA1 | -14,86 | 10,83 |
| IRF1 | -11,07 | 10,29 |
| **POU2F2** | -9,78 | 10,14 |

**Table S4:** Top 15 of the most influential TFs in CoRegNet using RNA-seq single cell from isolated fibroblasts

| ID | influence in OA | influence in RA |
| --- | --- | --- |
| GATA3 | -75.16 | 311,44 |
| **STAT1** | -119.64 | 247,55 |
| RELB | -43.02 | 230,13 |
| PML | -40.45 | 192,88 |
| **BATF** | -61.78 | 188,02 |
| FOXM1 | -70.34 | 181,39 |
| BCL11A | 2.43 | 180,66 |
| NFKB2 | -27.25 | 179,69 |
| **POU2AF1** | -54.44 | 172,22 |
| TOX | -44.00 | 165,78 |
| TNFAIP3 | -50.21 | 165,56 |
| **IRF4** | -50.16 | 160,56 |
| PRDM1 | -50.11 | 159,78 |
| **LEF1** | -51.79 | 150,96 |
| XBP1 | -45.31 | 146,24 |

**Table S5** : TOP 20 of the most active TFs in ISMARA using synovial tissue

| Motif | Z-score |
| --- | --- |
| **BATF** | 6.90 |
| RELA | 6.59 |
| EPAS1_BCL3 | 6.09 |
| DLX4_HOXD8 | 6.07 |
| ETV1_ERF_FEV_ELF1 | 5.16 |
| RFX3_RFX2 | 5.07 |
| REL | 5.06 |
| NFIX_NFIB | 4.93 |
| RARG | 4.90 |
| TFDP1 | 4.89 |
| POU5F1_POU2F3 | 4.82 |
| NR3C1 | 4.71 |
| POU3F3_POU3F4 | 4.67 |
| MEIS1 | 4.66 |
| **NFKB1** | 4.59 |
| RUNX1_RUNX2 | 4.50 |
| RELB | 4.50 |
| IRF2_STAT2_IRF8_IRF1 | 4.48 |
| GCM1 | 4.46 |
| FOSL2_SMARCC1 | 4.44 |

**Table S6** : TOP 20 of the most active TFs in dorothea using synovial tissue

| ID | activity in RA |
| --- | --- |
| RELA | 6,08 |
| NFKB1 | 5,63 |
| **STAT1** | 5,22 |
| **POU2F2** | 5,19 |
| IRF1 | 4,79 |
| ETS1 | 4,67 |
| TBX21 | 4,60 |
| RELB | 4,26 |
| REL | 4,06 |
| **BATF** | 3,88 |
| **IRF4** | 3,83 |
| SPIB | 3,71 |
| LYL1 | 3,68 |
| STAT2 | 3,67 |
| FOXL2 | 3,52 |
| IKZF1 | 3,24 |
| SPI1 | 3,23 |
| BCL11A | 3,22 |
| NFYB | 3,09 |
| ARID3A | 3,08 |

**Table S7:** TOP 20 of the most active TFs in ISMARA using microarray of isolated fibroblasts

| **Motif** | **Z_score** |
| --- | --- |
| HOXB6_PRRX2 | 4.87 |
| KLF16_SP2 | 3.86 |
| IRF3 | 3.63 |
| AHR_ARNT2 | 3.57 |
| RUNX1_RUNX2 | 3.41 |
| PBX3 | 3.24 |
| NFE2L1 | 3.14 |
| TBX1 | 2.85 |
| MAFK | 2.84 |
| IRF9 | 2.82 |
| IRF6_**IRF4**_IRF5 | 2.69 |
| IRF2_STAT2_IRF8_IRF1 | 2.68 |
| E2F3 | 2.67 |
| POU3F3_POU3F4 | 2.63 |
| EZH2 | 2.59 |
| UCACAUU | 2.54 |
| UCCAGUU | 2.52 |
| CUX2 | 2.52 |
| PPARA | 2.33 |
| TFAP4_MSC | 2.33 |

**Table S8 :** TOP 30 of the most active TFs in Dorothea using microarray of isolated fibroblasts.

| ID | activity |
| --- | --- |
| STAT2 | 7,68 |
| RFX5 | 5,71 |
| RFXANK | 5,64 |
| RFXAP | 5,64 |
| GRHL2 | 4,91 |
| **IRF4** | 4,68 |
| TFAP4 | 4,32 |
| PRDM14 | 4,08 |
| TCF12 | 4,01 |
| **STAT1** | 3,87 |
| EGR1 | 3,66 |
| MEF2A | 3,56 |
| IRF2 | 3,52 |
| E2F4 | 3,49 |
| MTA2 | 3,36 |
| THAP1 | 3,35 |
| IRF3 | 3,34 |
| EPAS1 | 3,28 |
| MTA1 | 3,22 |
| NFATC1 | 3,19 |
| MAFG | 3,18 |
| KLF9 | 3,17 |
| E2F1 | 3,17 |
| ELF1 | 3,13 |
| NR4A1 | 3,12 |
| MYOD1 | 3,12 |
| SREBF2 | 3,09 |
| ZNF384 | 3,05 |
| BACH2 | 3,05 |

**Table S9:** TOP 30 of the most active TFs in Dorothea using RNA-seq single cell of isolated fibroblasts

| ID | activity |
| --- | --- |
| RFXANK | 5,86 |
| RFXAP | 5,86 |
| RFX5 | 5,54 |
| PRDM14 | 4,76 |
| STAT2 | 3,92 |
| GRHL2 | 3,19 |
| THAP11 | 3,17 |
| MAFG | 3,12 |
| **STAT1** | 3,10 |
| MEIS1 | 3,02 |
| TBX21 | 2,92 |
| **IRF4** | 2,83 |
| EPAS1 | 2,81 |
| GATA6 | 2,78 |
| IRF2 | 2,78 |
| T | 2,59 |
| NR2F1 | 2,52 |
| FLI1 | 2,48 |
| ESR1 | 2,46 |
| IKZF1 | 2,40 |
| **BATF** | 2,38 |
| SRF | 2,36 |
| SOX9 | 2,35 |
| SP3 | 2,34 |
| SPI1 | 2,32 |
| SPIB | 2,28 |
| MEF2B | 2,24 |
| ETV4 | 2,18 |
| IRF1 | 2,13 |

**Table S10**: DE apoptosis markers.

| ID | logFC | P-value | Regulation type |
| --- | --- | --- | --- |
| GAS6 | 3.93 | 9.99e-32 | negative |
| SFRP1 | 2.49 | 1.23e-15 | negative and positive |
| GSTM1 | -3,35 | 1,41E-24 | positive |
| BCL2L10 | -2,89 | 1,06E-17 | positive |
| BTG2 | -2,47 | 2E-15 | positive |
| GPX3 | -2,33 | 3,95E-14 | positive |
| EREG | -2,15 | 2,89E-12 | positive |
| CLU | -2,00 | 5,04E-11 | positive |
| TIMP1 | -1,95 | 1,55E-10 | positive |
| TXNIP | -1,68 | 2,6E-08 | positive |
| CASP3 | -1,50 | 5,36E-07 | positive |

**Table S11**: DE migration markers.

| ID | LogFC | P-value | Regulation type |
| --- | --- | --- | --- |
| THBS1 | 1.67 | 3.29e-08 | positive |
| MMP14 | 1,38 | 5,28e-06 | positive |

**Table S12**: DE proliferation markers.

| ID | logFC | p-value | regulation type |
| --- | --- | --- | --- |
| PPARG | -1.609 | 1.11e-07 | negative |
| CCNB1 | 1.50 | 1.94e-06 | positive |
| CD74 | 2.41 | 8.96e-15 | positive |
| GAS6 | 3.93 | 9.99e-32 | positive |
| IGF1 | 2.85 | 1.90e-19 | positive |
| PDGFRB | 1.98 | 8.82e-11 | positive |
| SPHK1 | 2.40 | 3.76e-14 | positive |

**Table S13:** Differentially expressed pro-inflammation markers:

| HGNC symbol | logFC | P-Value |
| --- | --- | --- |
| NOD2 | 1,53 | 2,4E-06 |
| IL15RA | 1,59 | 3,53E-07 |
| IL10RA | 1,90 | 9,36E-09 |
| RASGRP1 | 2,05 | 8,24E-11 |
| KCNJ2 | 2,15 | 3,31E-11 |
| SLC1A2 | 2,20 | 3,59E-12 |
| SPHK1 | 2,40 | 3,76E-14 |
| LAMP3 | 2,55 | 1,61E-14 |
| RGS1 | 2,87 | 6,03E-16 |
| GABBR1 | 2,97 | 1,31E-19 |
| STAB1 | 3,25 | 7,66E-23 |
| CXCL9 | 3,33 | 6,43E-24 |
| F3 | 3,45 | 2,63E-26 |
| TACR3 | 3,60 | 3,55E-25 |
| IL6 | 4,18 | 3,07E-32 |
| CD48 | 4,67 | 5,86E-37 |
| CXCL11 | 4,76 | 9,36E-37 |
| CXCL10 | 5,49 | 2,11E-48 |

**Table S14:** Summary of the datasets characteristics

|  | **Synovial tissue** | **Microarray of fibroblasts** | **RNA-seq SC of Fibroblasts** |
| --- | --- | --- | --- |
| **Number of samples** | 79 | 37 (3 OA patients and 3 RA patients) from the latest stages of RA | 384 fibroblasts (two with OA and two with RA, 96 cells from each patient) |
| **number of control (OA)** | 26 | 15 | 192 cells |
| **sex(male/female)** | 4/22 for OA  8/25 for RA | 5/10 for OA (1 / 2)  6/16 for RA ( 1 / 2) | 0/192 for OA (0/2)  0/192 for RA (0/2) |
| **age** | 71.0 ± 1.4 for OA  57.0 ± 2.7 for RA | 59- 78- 86 for OA  51- 79-48 for RA | 61 and 70 for OA   61 and 67  for RA |
| **origin** | “tissues were collected from joint replacement surgery in OA patients and synovectomy surgery in RA patients, normal synovium was collected early post mortem from macroscopically normal knee joints.”  No information for RA and OA samples | -all from knee for OA.  -15 samples from knee and 7 from metacarpophalangeal joint for RA. | -all from knee for OA.  -96 cells from elbow joint and 96 cells from knee joint. |
| **prior treatement for OA** | NSAIDs (*n* = 16)  None (*n* = 10) | No information provided | No information provided |
| **prior treatement for RA** | Prednisolone (*n* = 23)  Methotrexate (*n* = 18)  Sulfasalazine (*n* = 5)  Chloroquine (*n* = 2)  Leflunomide (*n* = 2)  Cyclosporine (*n* = 1)  Gold (*n* = 1)  NSAIDs (*n* = 22) | No information provided. | No information provided |
| **Disease duration (years)** | 7.0 ± 1.3 for OA patients  12.5 ± 2.0 for RA patients | No exact duration provided. The authors state that the gene expression data is limited to synovial specimens from the latest stages of RA and therefore represent changes in chronic late-stage RA. | No exact duration provided. The authors state that the gene expression data is limited to synovial specimens from the latest stages of RA and therefore represent changes in chronic late-stage RA. |

**Table S15:** Results of the statistical analysis and comparison of the three datasets used in the study for the sex of the patients:

| - synovial tissue dataset/ Microarray of synovial fibroblasts in RA samples: | X-squared = 0, df = 1, p-value = 1 (Chi2) /p-value = 1 (Fisher) |
| --- | --- |
| - synovial tissue dataset/ Microarray of synovial fibroblasts in OA samples: | X-squared = 2.9391e-31, df = 1, p-value = 1(Chi2)/ p-value = 0.4461 (Fisher) |
| - synovial tissue dataset/ RNA-seq SC of synovial fibroblasts in RA samples: | X-squared = 7.6532e-30, df = 1, p-value = 1(Chi2) /p-value = 1 (Fisher) |
| - synovial tissue dataset/ RNA-seq SC of synovial fibroblasts in OA samples: | X-squared = 4.4258e-30, df = 1, p-value = 1 (Chi2) / p-value = 1 (Fisher) |
| - Microarray of synovial fibroblasts / RNA-seq SC of synovial fibroblasts in RA samples: | X-squared = 5.1358e-32, df = 1, p-value = 1 (Chi2) / p-value = 1 (Fisher) |
| - Microarray of synovial fibroblasts /RNA-seq SC of synovial fibroblasts in OA samples: | X-squared = 5.1358e-32, df = 1, p-value = 1 (Chi2) / p-value = 1 (Fisher) |

**S16:** Results of the statistical analysis and comparison of the three datasets used in the study for the age of the patients:

| - Synovial tissue dataset/ Microarray of synovial fibroblasts in RA samples: | t = 0.36428, df = 2.5212, p-value = 0.744 (t-test)/W = 31.5, p-value = 0.8407 (Wilcoxon test) |
| --- | --- |
| - Synovial tissue dataset/ Microarray of synovial fibroblasts in OA samples: | t = 0.20108, df = 2.1832, p-value = 0.8578 (t-test)/W = 18, p-value = 0.5361 (Wilcoxon test) |
| - Synovial tissue dataset/ RNA-seq SC of synovial fibroblasts in RA samples: | t = 1.8484, df = 5.0537, p-value = 0.1232 (t-test)/W = 15, p-value = 0.4516 (Wilcoxon test) |
| - Synovial tissue dataset/ RNA-seq SC of synovial fibroblasts in OA samples: | t = -1.4944, df = 1.303, p-value = 0.332 (t-test)/W = 26.5, p-value = 0.1572 (Wilcoxon test) |
| - Microarray of synovial fibroblasts / RNA-seq SC of synovial fibroblasts in RA samples: | t = 0.45232, df = 2.3465, p-value = 0.6895 (t-test)/W = 2, p-value = 0.8 (Wilcoxon test) |
| - Microarray of synovial fibroblasts /RNA-seq SC of synovial fibroblasts in OA samples: | t = -0.96173, df = 2.8869, p-value = 0.4096 (t-test)/W = 4, p-value = 0.8 (Wilcoxon test) |

*
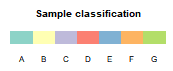
*


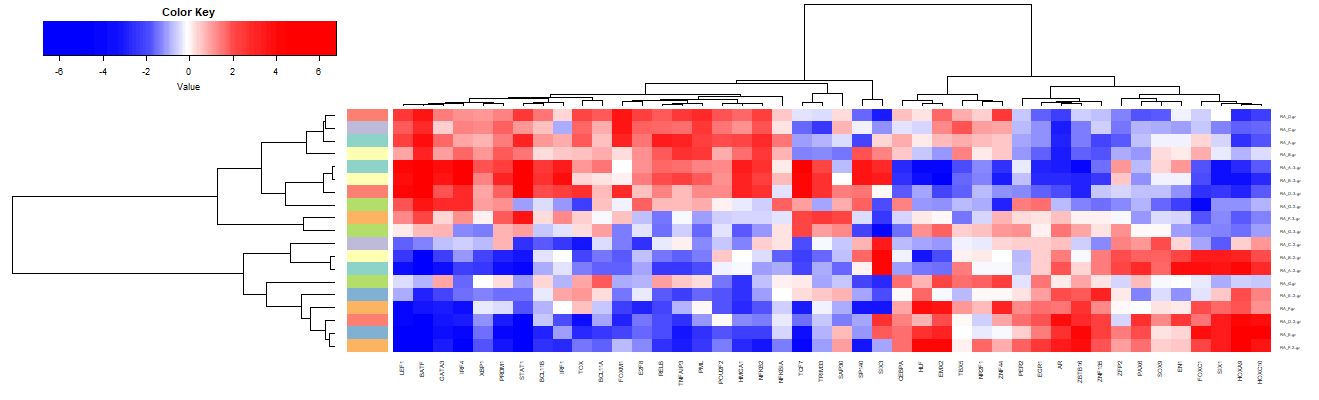


**Figure S1 :** Heatmap showing the TFs influence profiles of the seven fibroblasts’ subpopulations in RA patients. The samples and genes clustering is performed using Pearson correlation.


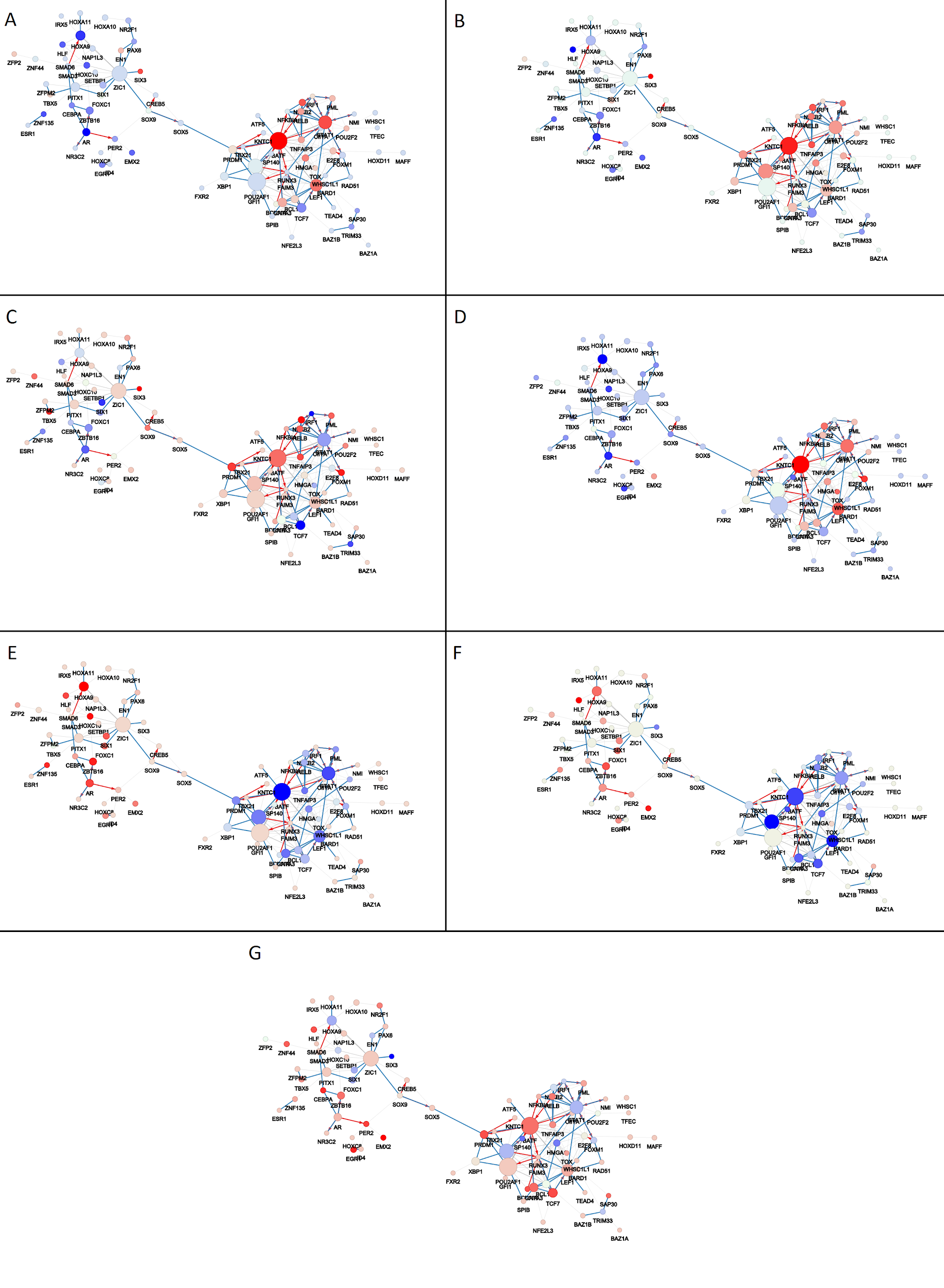


**Figure S2 :** Co-regulatory networks showing the seven fibroblasts subpopulation TF influence profiles in RA patients.

*
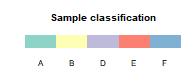
*


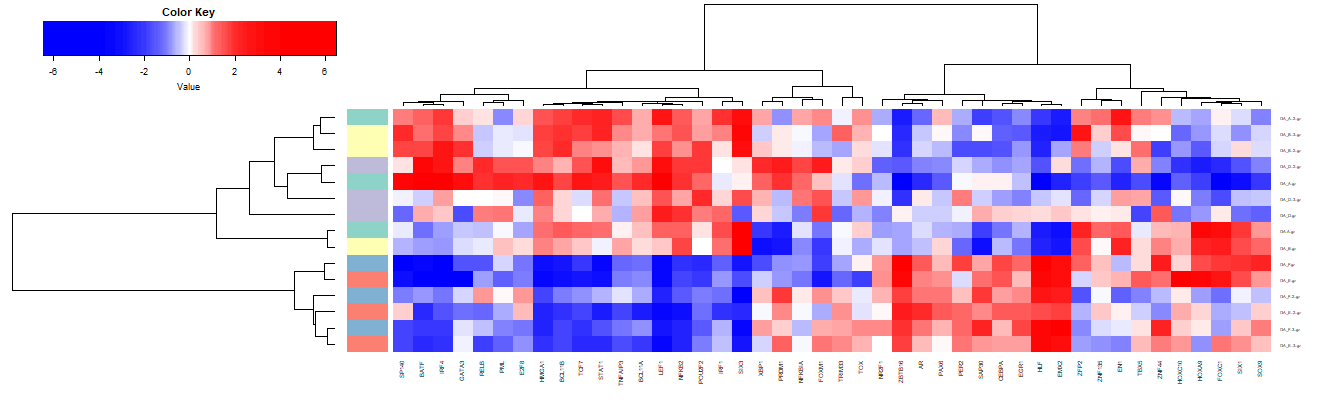


**Figure S3 :** Heatmap showing the TFs influence profiles of the five fibroblasts subpopulation in OA patients. The samples and genes clustering is performed using Pearson correlation.


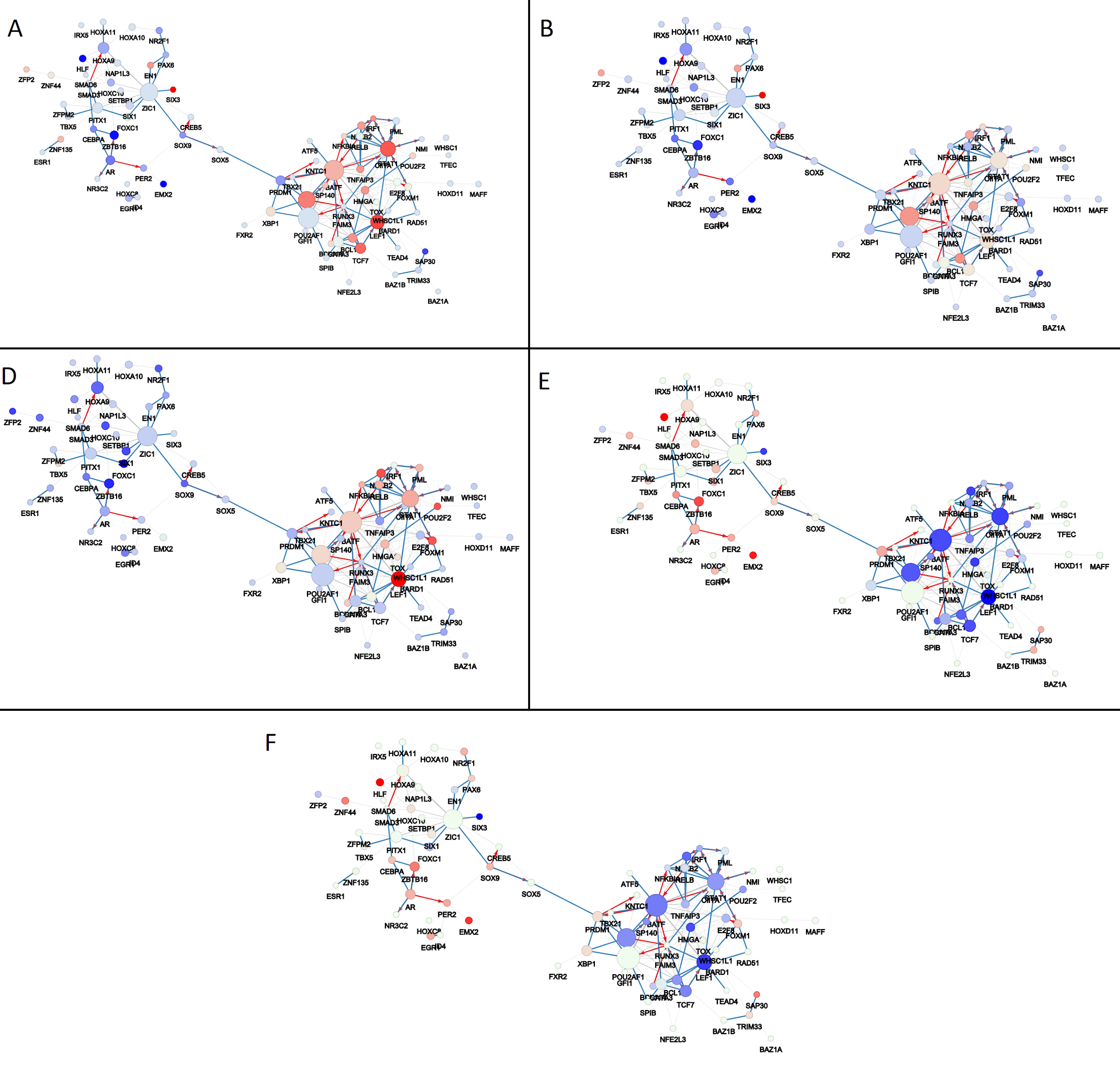


**Figure S4:** Co-regulatory networks showing the five fibroblasts subpopulation profiles in OA patients.


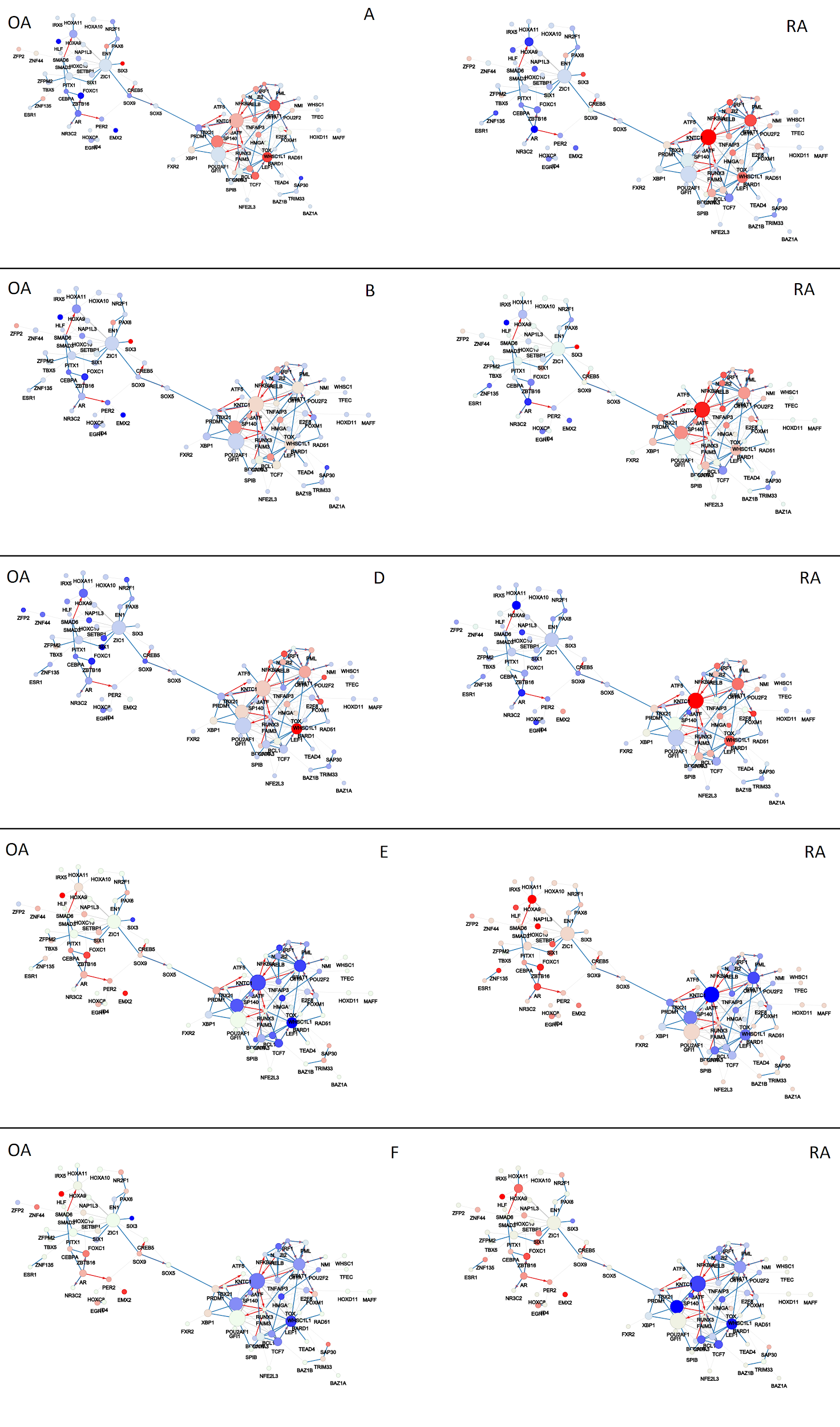


**Figure S5**: Comparison between the fibroblasts subpopulations profiles between RA and OA patients.
